# PRMT5 inhibitors on the (myeloma) road

**DOI:** 10.18632/oncotarget.26392

**Published:** 2018-11-30

**Authors:** Annamaria Gulla, Teru Hideshima, Kenneth C. Anderson

**Affiliations:** Jerome Lipper Multiple Myeloma Center, Medical Oncology, Dana Farber Cancer Institute, Boston, MA, USA

**Keywords:** PRMT5, myeloma, epigenetics, methylation, PTM

The introduction of novel therapies targeting either proteasome overload, the multiple myeloma (MM) microenvironment and/or immune effectors has dramatically improved the outcome of MM patients; nonetheless MM ultimately relapses and remains a deadly disease [1]. The lack of common genetic alterations leading to relapse and refractory MM prompted the study of different mechanisms underlying tumor progression; and advanced high-throughput technologies provided the means to study the complex architecture of MM cells [1]. Along with genetic heterogeneity, it is now clear that a new layer of modifications in the epigenetic machinery (such as DNA methylation, histone post translational modifications (PTMs) or non-coding RNAs) impacts MM cell biology and pathogenesis; moreover, aberrant non-histone PTMs fine tune protein-protein interactions and cell signaling cascades promoting tumor cell survival [2, 3]. Among several PTMs, recent evidence has highlighted the critical role of methylation in regulating a wide range of cellular processes. Specifically, aberrant methylation occurring on arginine residues, mediated by protein arginine methyltransferases (PRMTs), has recently gained attention for its role in cancer initiation and progression [4]. Among 9 members of the PRMTs family, many have been described to methylate both histone and non-histone substrates; by bridging epigenetic control and signal transduction, they may regulate essential cellular processes and determine the functional diversity of proteins. Aberrant expression of PRMTs has been found in several cancers, including hematologic malignancies [5, 6].

We recently provided evidence of the oncogenic role of PRMT5 in MM [7]. PRMT5 is the main type II PRMT which catalyzes the symmetric transfer of up to two methyl groups to arginine residues; and increased PRMT5 activity exerts an oncogenic effect in several types of cancer, promoting malignant transformation and tumor progression [8]. Analysis of RNA-sequencing data from 308 clinically-annotated patients with newly diagnosed MM showed that PRMT5 was upregulated and associated with poor clinical outcome [7]. PRMT5 upregulation was correlated with disease progression; and importantly, either PRMT5 genetic knockdown or pharmacological inhibition with a small molecule EPZ015666 inhibited growth and survival of MM cells, even within the protective bone marrow microenvironment [7].

We went on to delineate its mechanism of action in MM. PRMT5 is a versatile enzyme whose activity accounts for concomitant effects of histone and non-histone proteins targets, both in the cytoplasm and the nucleus. Among its functions, it induces transcriptional repression *via* the preferential methylation of H4R3 over H3R8, and controls the expression of key survival genes by cooperating with specific ATP-dependent chromatin remodelers, co-repressors, and co-activators. Its oncogenic activity extends beyond epigenetic effects, since PRMT5 also targets multiple non-histone proteins including p53, E2F1 and Sm proteins; as well as regulates splicing machinery, as critical component of the methylosome [8]. In our study we focused on its activity on non-histone proteins in order to identify its role in regulation of biologically important MM signaling cascades. We found that inhibition of PRMT5 decreased NF-κB signaling in MM cells, which mediates growth, survival, and drug resistance in MM cells, as well as regulates expression of adhesion molecules and transcription of cytokines in the BM milieu. By using mass spectrometry, we identified the tripartite motif-containing protein 21 TRIM21 as a new PRMT5-partner. Our data showed that PRMT5-mediated methylation of TRIM21 has a key regulatory function of NF-κB signaling in MM cells that occurs through the inhibition of autophagic proteolysis of IKKβ [7].

**Figure 1:**
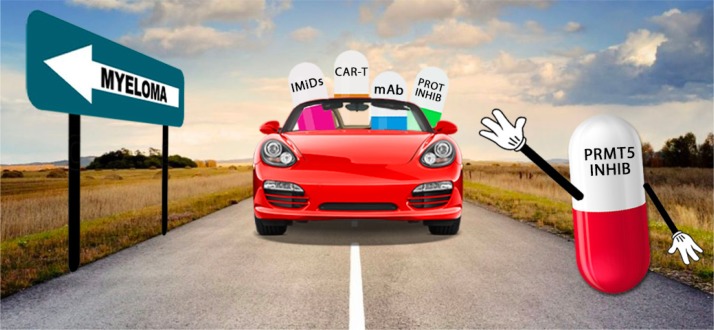
PRMT5 inhibitors are promising new agents in anti-myeloma armamentarium

Our study demonstrates the functional significance of PRMT5 and validates the anti-MM activity of EPZ015666 in murine xenograft models of human MM, providing the framework for clinical trials targeting PRMT5 in relapsed MM (Figure 1). To achieve this goal, ongoing studies are evaluating potential additional roles of PRMT5 in MM, including transcriptional control of gene expression *via* histone methylation or regulation of splicing machinery. These studies may not only delineate additional biologic sequelae of PRMT5 inhibition, but also identify potential biomarkers to inform clinical practice. Importantly, efforts are underway to develop a more potent and selective PRMT5 inhibitor for clinical translation, alone and in scientifically-informed combination therapies. Ultimately, targeting PRMT5 and its broad biologic sequelae in tumor cells represents a promising novel therapeutic strategy to improve patient outcome in patients with MM and other cancers.
